# Supplementary material for: Interobserver Agreement of PD-L1/SP142 Immunohistochemistry and Tumor-Infiltrating Lymphocytes (TILs) in Distant Metastases of Triple-Negative Breast Cancer: A Proof-of-Concept Study. A Report on Behalf of the International Immuno-Oncology Biomarker Working Group
Source: Cancers (Basel). 2021 Sep 29;13(19):4910. doi: 10.3390/cancers13194910 (PMC8507620; doi:10.3390/cancers13194910)
Supplement: Supplementary file 1 [file cancers-13-04910-s001.zip › cancers-1366162-SI.zip!/cancers-1366162-SI.pdf]

cancers

Supplementary Materials: Interobserver Agreement of PD-L1/SP142 Immunohistochemistry and Tumor-Infiltrating lymphocytes (TILs) in Distant Metastases of Triple-Negative Breast Cancer: A Proof-of-Concept Study. A Report on Behalf of the International Immuno-Oncology Biomarker Working Group

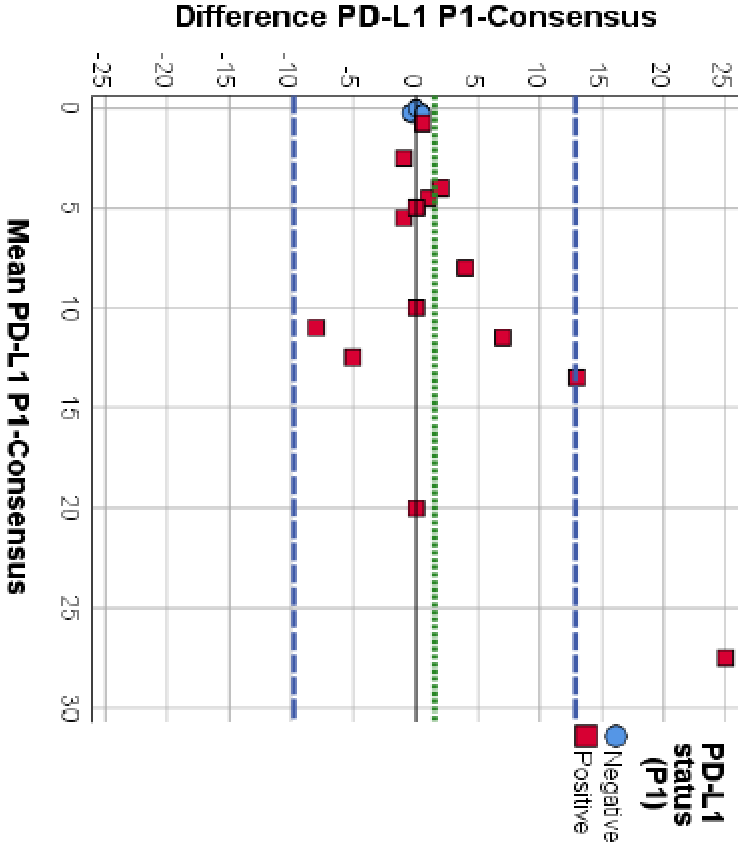

Supplementary Figure S1. *Cont.*

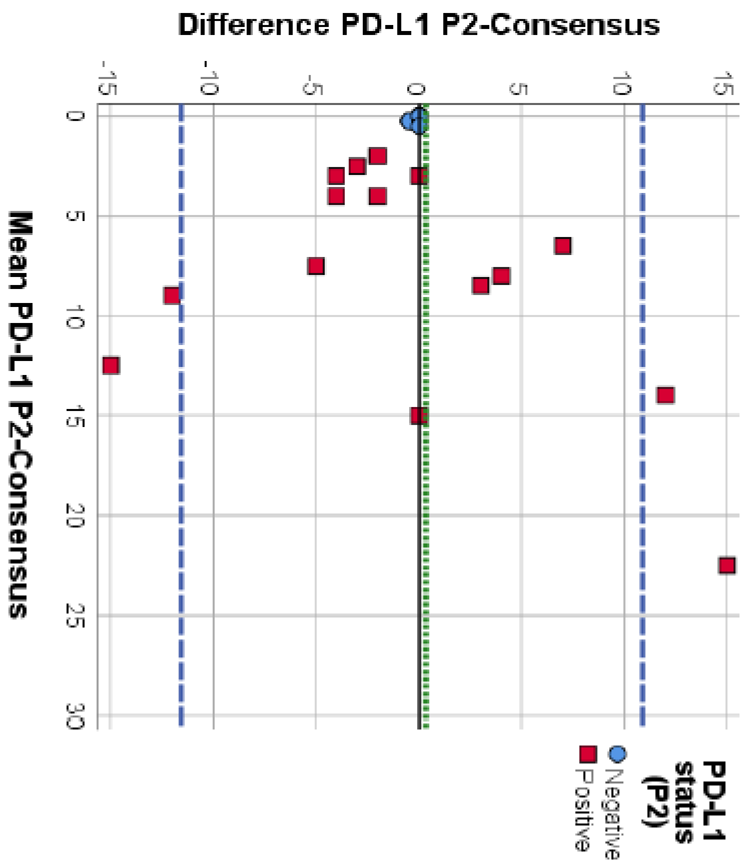

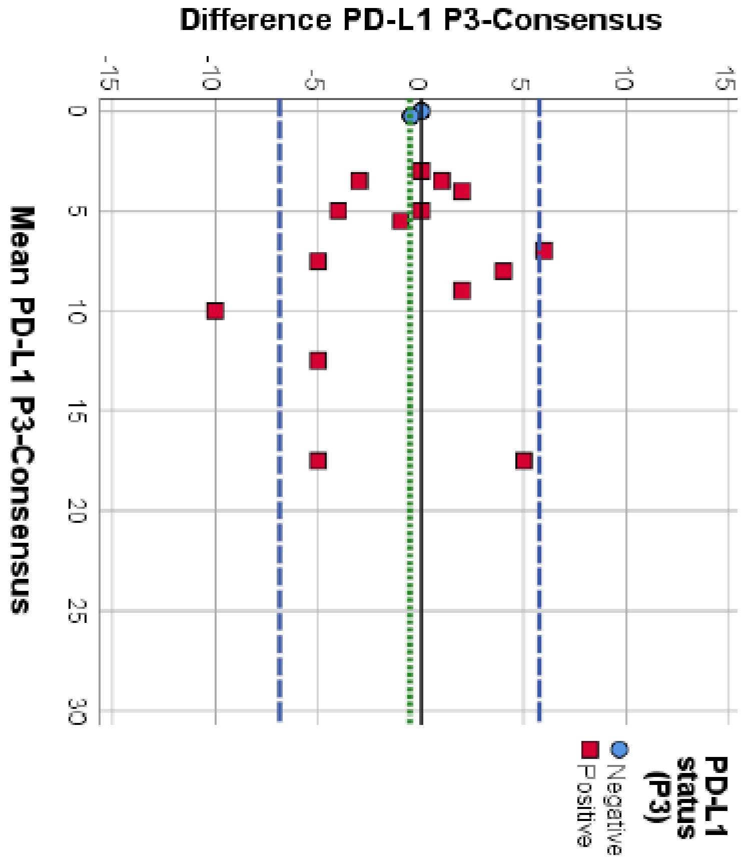

Supplementary Figure S1. *Cont.*

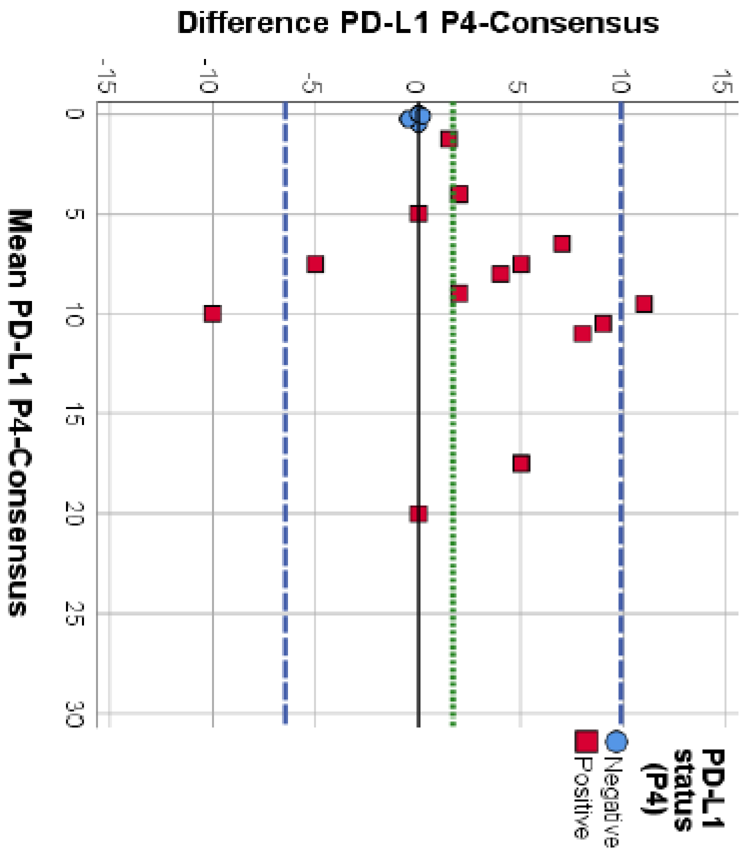

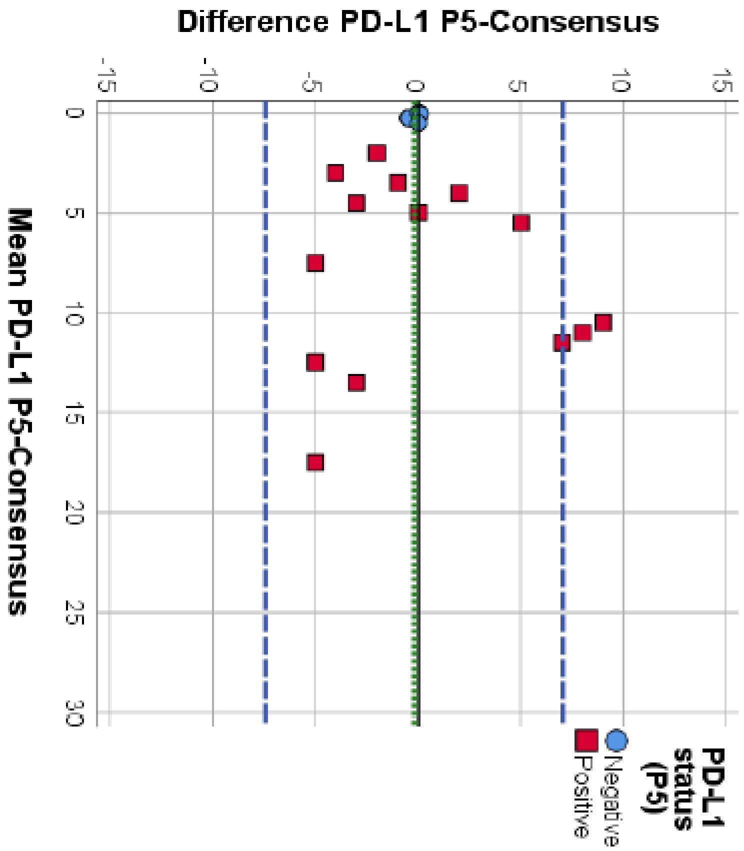

Supplementary Figure S1. *Cont.*

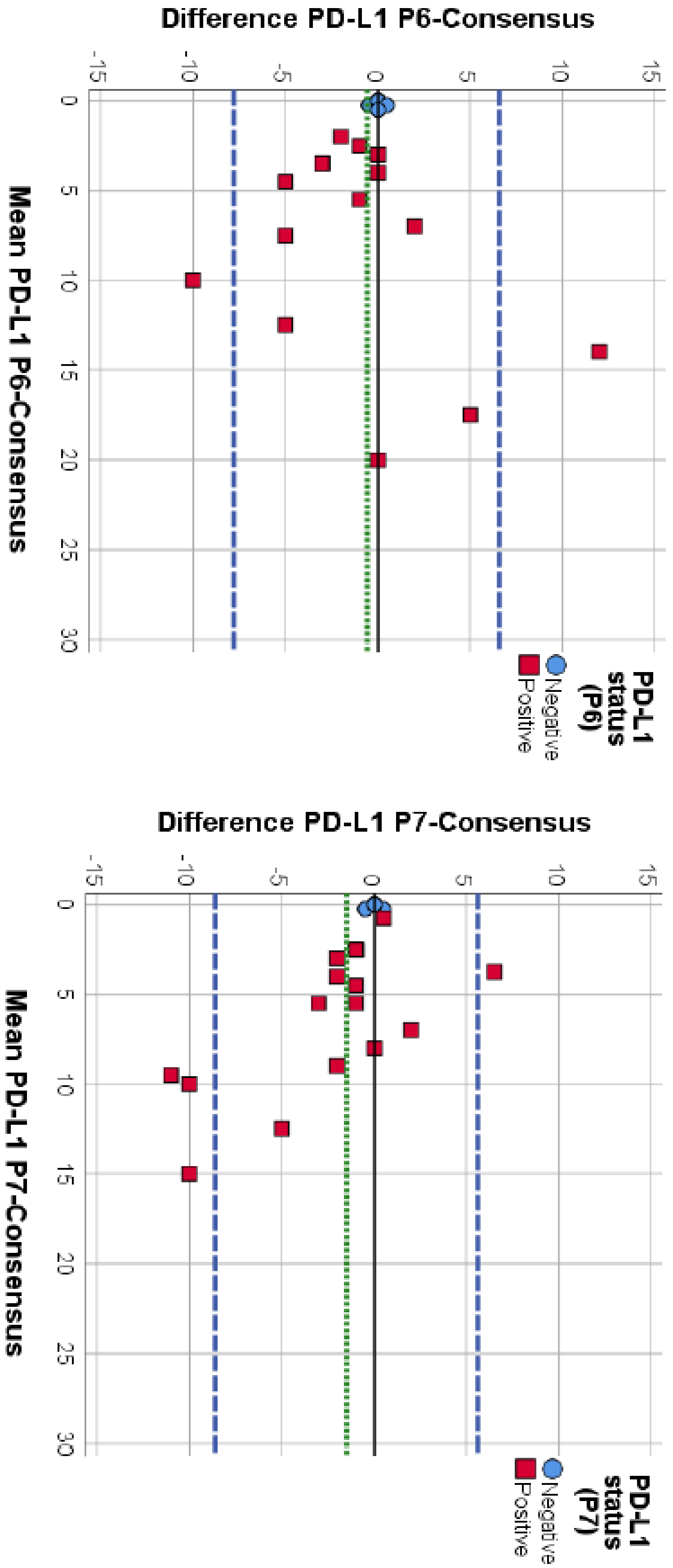

Supplementary Figure S1. *Cont.*

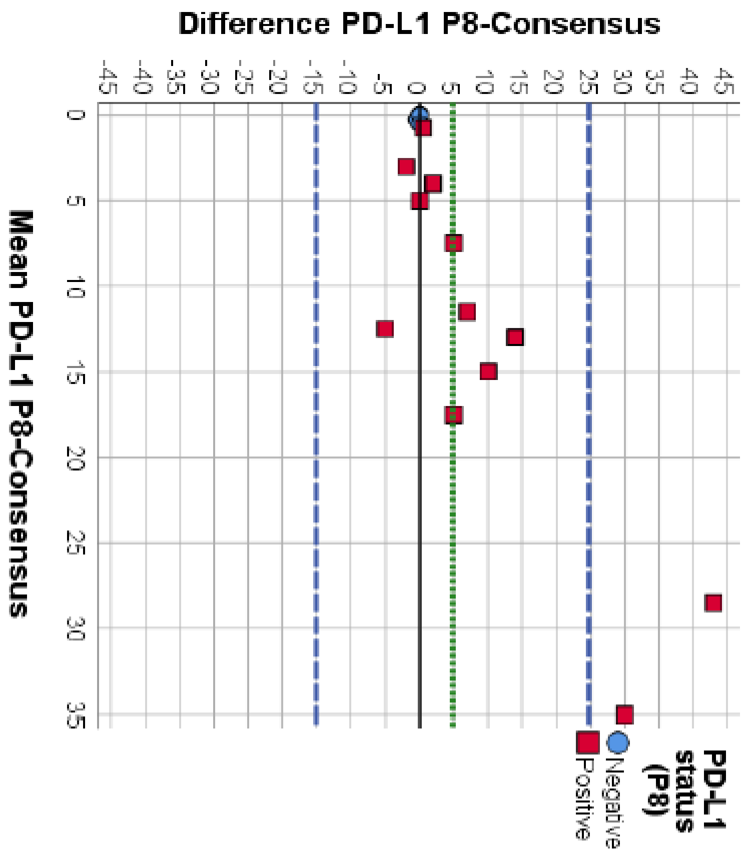

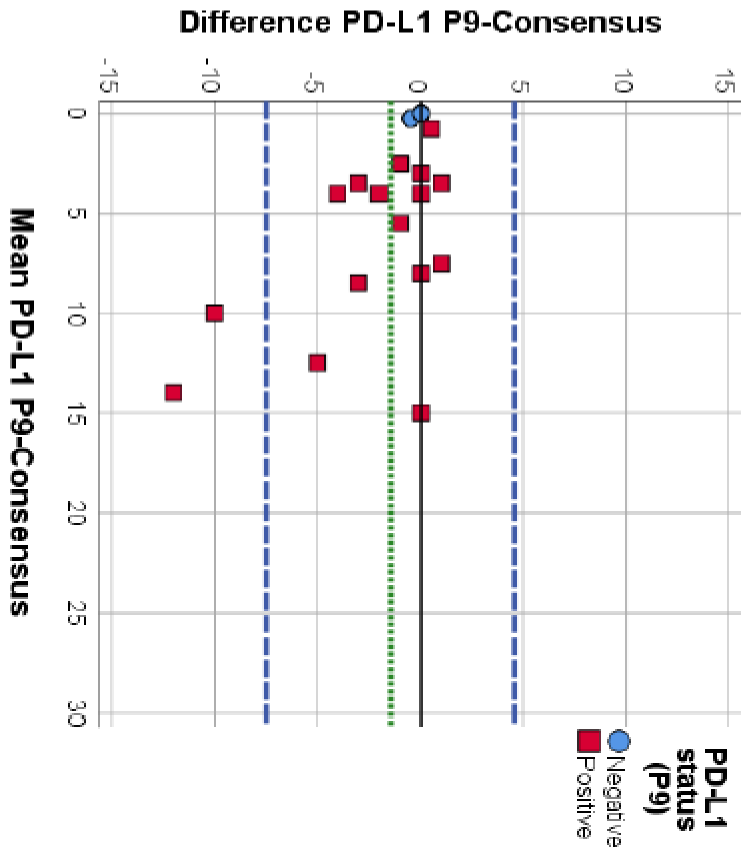

Supplementary Figure S1. *Cont.*

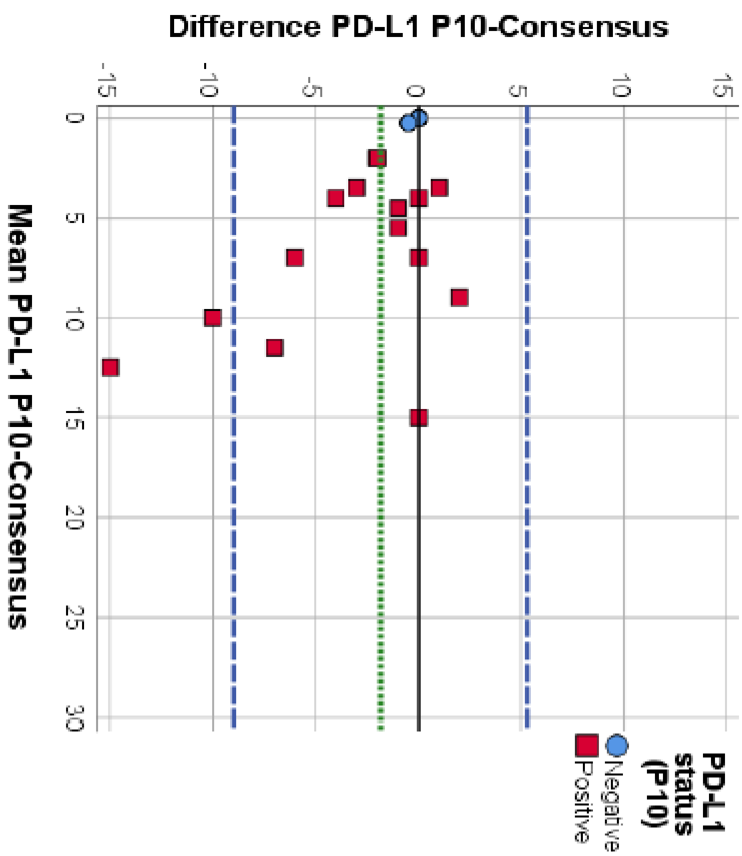

Supplementary Figure S1. Bland-Altman plots illustrating the variation in PD-L1/SP142 scores per pathologist in the proficiency set.

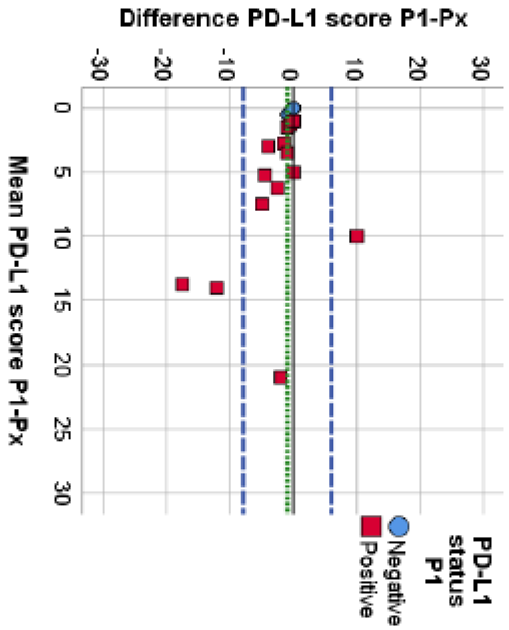

Supplementary Figure S2. *Cont.*

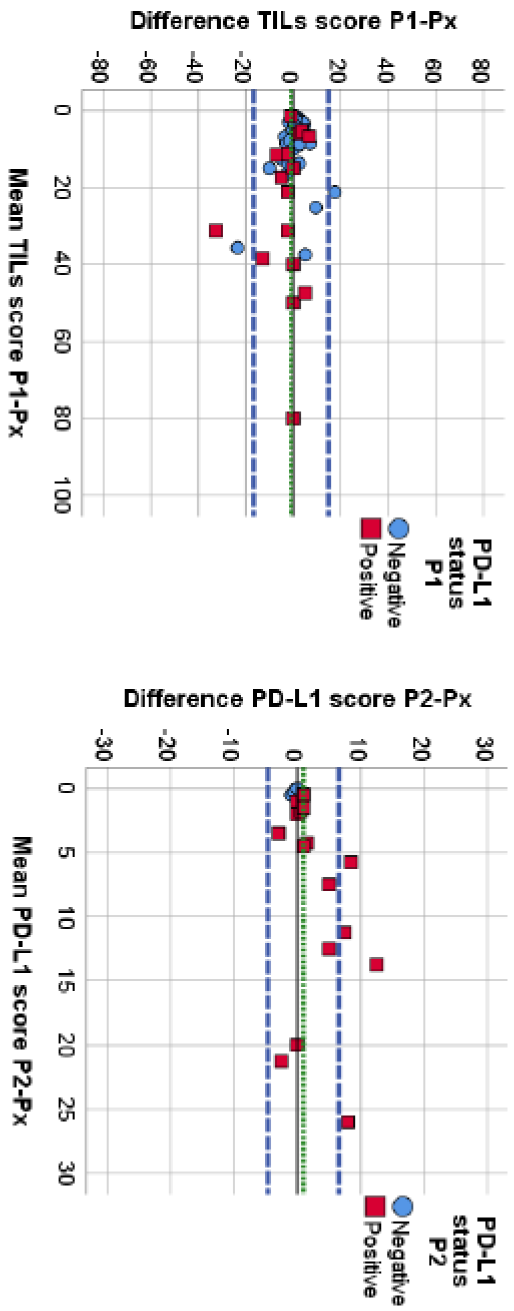

Supplementary Figure S2. *Cont.*

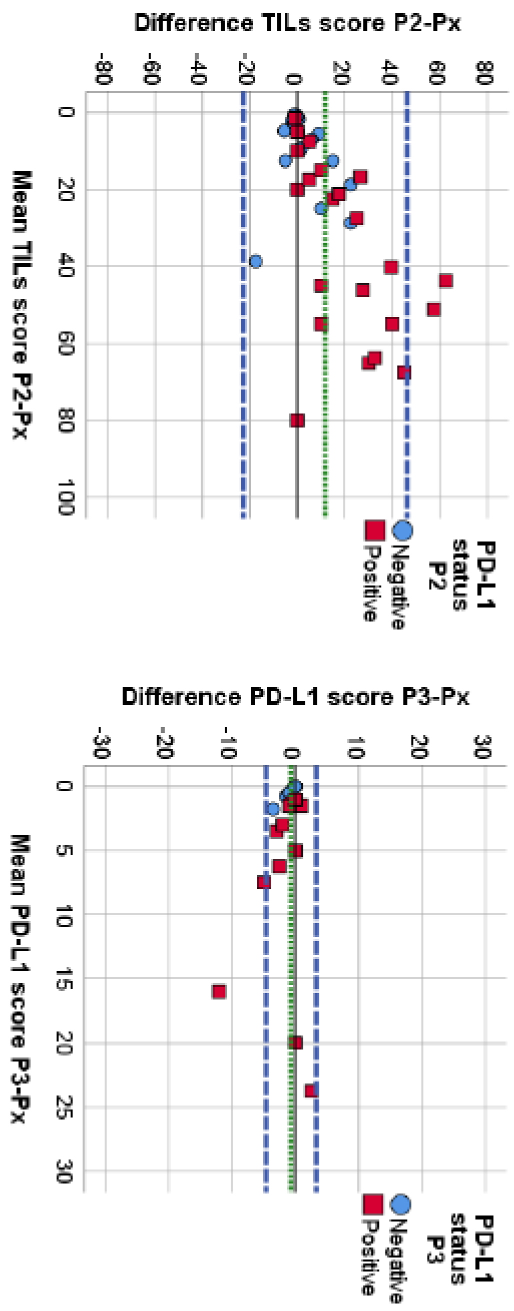

Supplementary Figure S2. *Cont.*

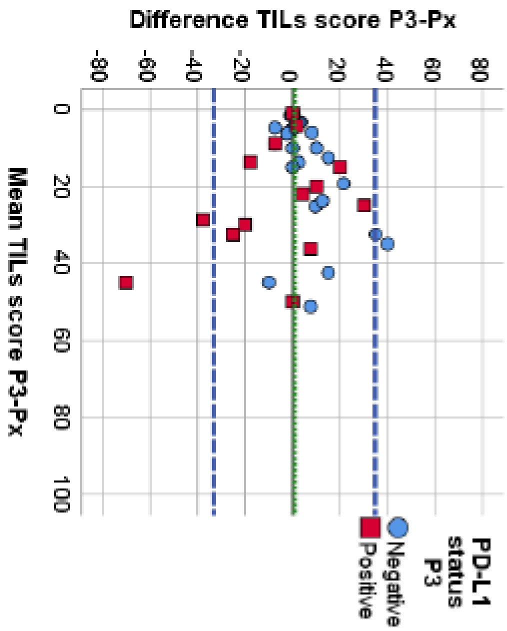

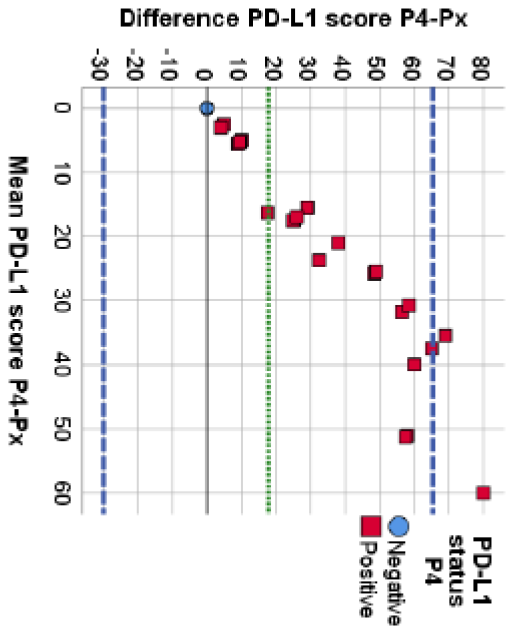

Supplementary Figure S2. *Cont.*

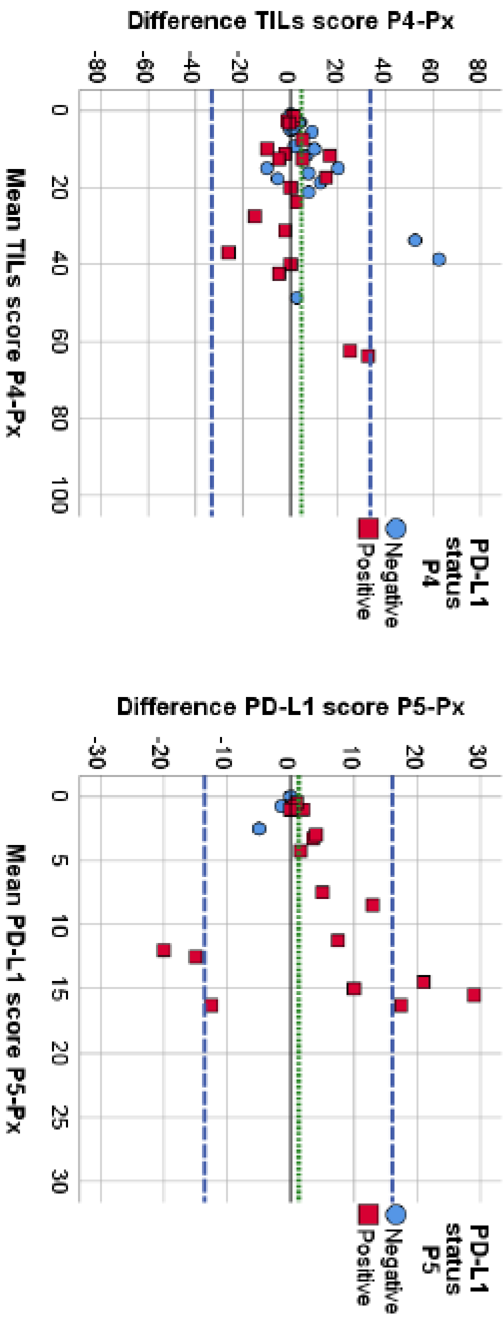

Supplementary Figure S2. *Cont.*

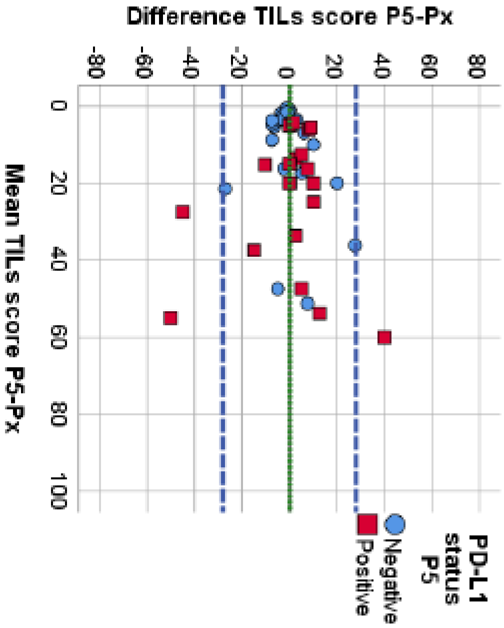

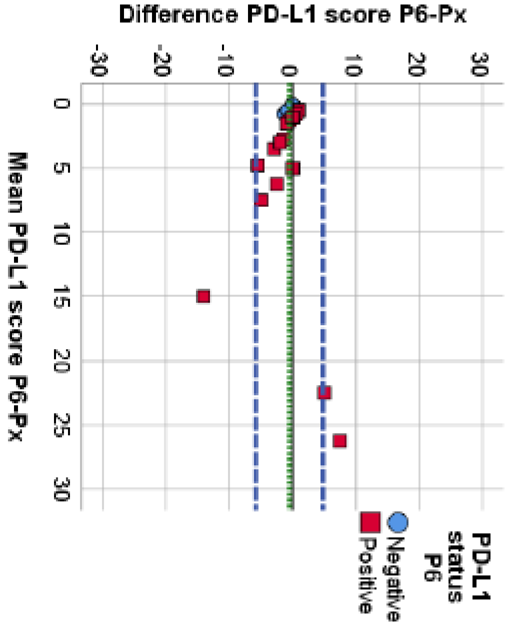

Supplementary Figure S2. *Cont.*

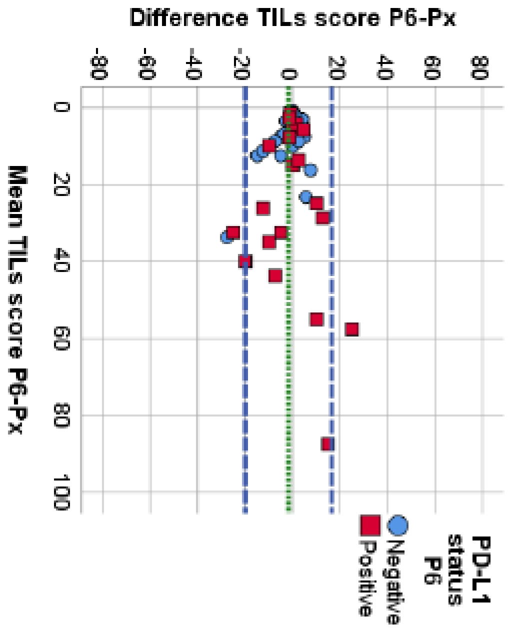

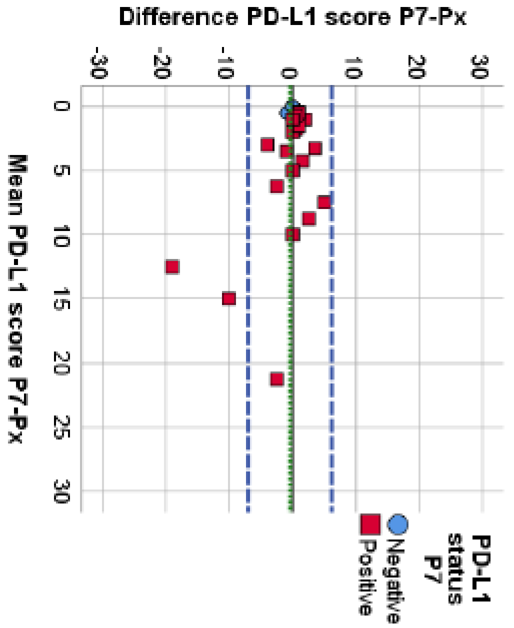

Supplementary Figure S2. *Cont.*

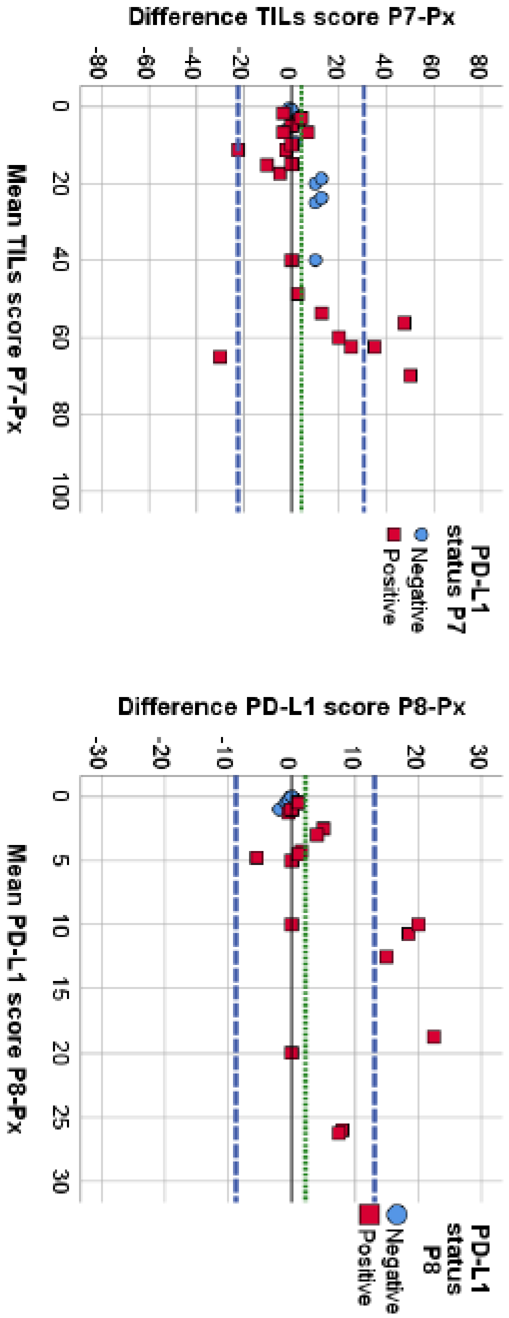

Supplementary Figure S2. *Cont.*

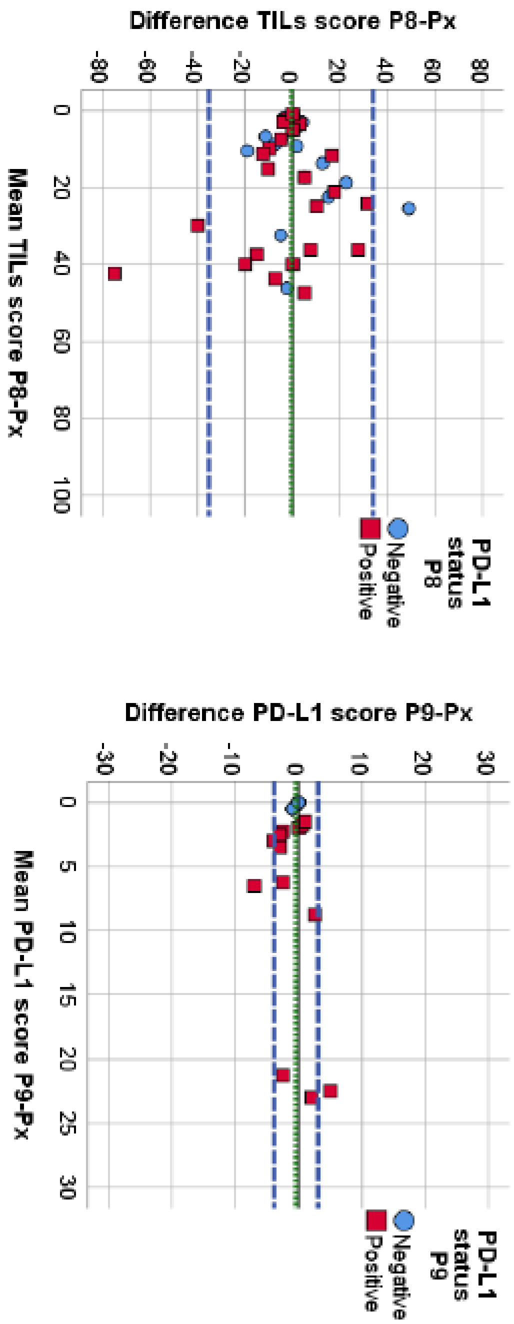

Supplementary Figure S2. *Cont.*

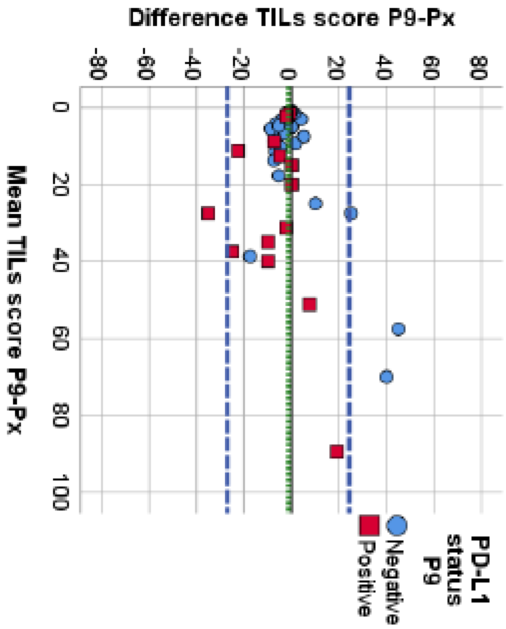

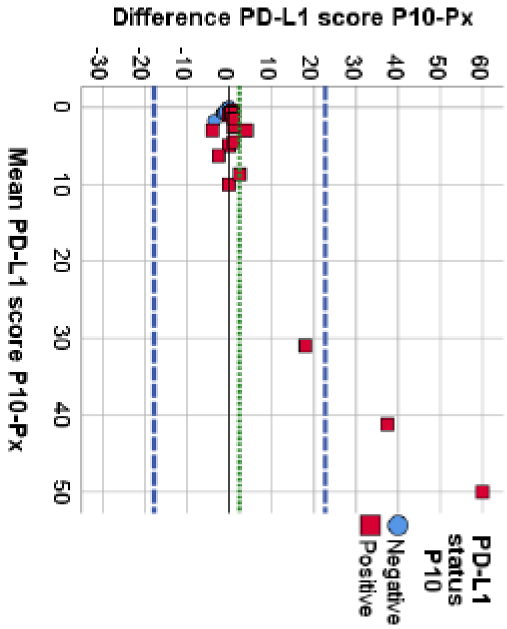

Supplementary Figure S2. *Cont.*

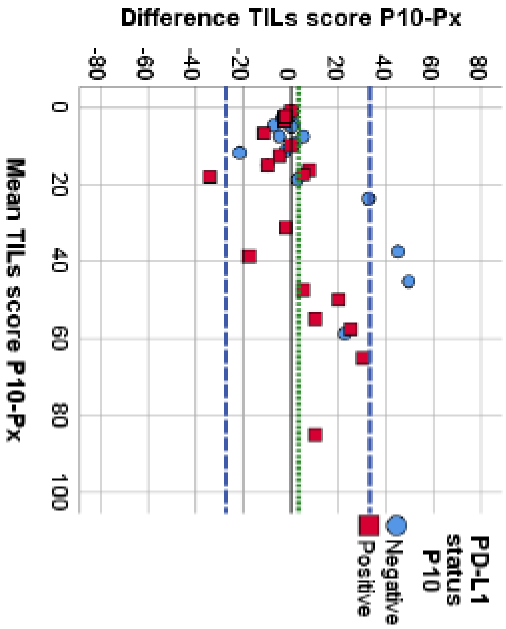

Supplementary Figure S2. Bland-Altman plots illustrating the variation in PD-L1/SP142 scores and TILs assessment per pathologist in the study set.

Supplementary Table S1. Descriptives of the PD-L1/SP142 assessment per pathologist in the study set.

PD-L1 / SP142	Min	Max	Mean	StDev	P5	P10	P25	P50 = median	P75	P90	P95
PD-L1 assessment P1 (%)	0	20	1.6	3.8	0	0	0	0	1	5	11.9
PD-L1 assessment P2 (%)	0	30	3.5	6.8	0	0	0	0	2	15	20
PD-L1 assessment P3 (%)	0	25	1.8	4.8	0	0	0	0	1	5	15
PD-L1 assessment P4 (%)	0	100	20.3	28.2	0	0	0	0	35	70	80
PD-L1 assessment P5 (%)	0	30	3.8	7.4	0	0	0	0	5	15	25
PD-L1 assessment P6 (%)	0	30	2.0	5.6	0	0	0	0	1	5	16.5
PD-L1 assessment P7 (%)	0	20	2.1	3.9	0	0	0	1	2	10	10
PD-L1 assessment P8 (%)	0	30	4.7	8.6	0	0	0	0	5	20	30
PD-L1 assessment P9 (%)	0	25	2.1	5.7	0	0	0	0	1	5	22
PD-L1 assessment P10 (%)	0	80	4.9	15.0	0	0	0	0	2.5	10	50
Median PD-L1 score Px (%)	0	22.5	2.5	5.4	0	0	0	0	1.8	7.5	21

Supplementary Table S2. Descriptives of the TILs assessment per pathologist in the study set.

TILs	Min	Max	Mean	StDev	P5	P10	P25	P50 = median	P75	P90	P95
TILs assessment P1 (%)	1	80	15	15.7	2	3	5	10	17.5	40	50
TILs assessment P2 (%)	0	90	28	29.1	0.5	1	2	20	45	80	85
TILs assessment P3 (%)	1	55	17	16.4	1	1	5	10	27.5	50	52.5
TILs assessment P4 (%)	1	80	19	19.8	2	3	5	12.5	25	51	73
TILs assessment P5 (%)	0	80	16	18.6	0	0	2	10	25	50	57.5
TILs assessment P6 (%)	1	95	14	19.1	1	1	4	5	20	35	65
TILs assessment P7 (%)	0	95	20	25.0	0	1	5	10	27.5	70	80
TILs assessment P8 (%)	0	50	15	16.4	1	1	1	5	30	40	50
TILs assessment P9 (%)	0	99	15	23.0	1	1	1	5	17.5	40	85
TILs assessment P10 (%)	1	90	19	25.8	1	1	1	5	30	70	75
Median TILs score Px (%)	1	80	16	17.9	1	1.5	3.5	8.5	20.5	47.5	50
