# Supplementary material for: Interobserver Agreement of PD-L1/SP142 Immunohistochemistry and Tumor-Infiltrating Lymphocytes (TILs) in Distant Metastases of Triple-Negative Breast Cancer: A Proof-of-Concept Study. A Report on Behalf of the International Immuno-Oncology Biomarker Working Group
Source: Cancers (Basel). 2021 Sep 29;13(19):4910. doi: 10.3390/cancers13194910 (PMC8507620; doi:10.3390/cancers13194910)
Supplement: Supplementary file 1 [file cancers-13-04910-s001.zip › cancers-1366162-tex.zip!/Definitions/cancers-1366162-g001.pdf]

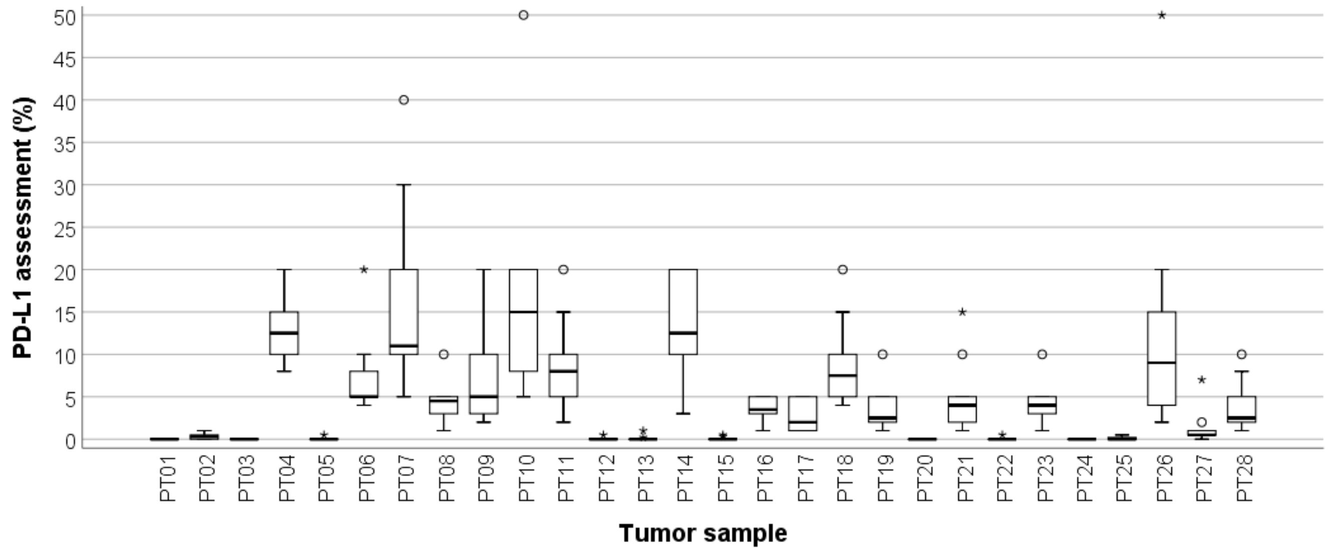
PD-L1 assessment (%)
0
5
10
15
20
25
30
35
40
45
50
PT01
PT02
PT03
PT04
PT05
PT06
PT07
PT08
PT09
PT10
PT11
PT12
PT13
PT14
PT15
PT16
PT17
PT18
PT19
PT20
PT21
PT22
PT23
PT24
PT25
PT26
PT27
PT28
Tumor sample
