# Supplementary material for: Interobserver Agreement of PD-L1/SP142 Immunohistochemistry and Tumor-Infiltrating Lymphocytes (TILs) in Distant Metastases of Triple-Negative Breast Cancer: A Proof-of-Concept Study. A Report on Behalf of the International Immuno-Oncology Biomarker Working Group
Source: Cancers (Basel). 2021 Sep 29;13(19):4910. doi: 10.3390/cancers13194910 (PMC8507620; doi:10.3390/cancers13194910)
Supplement: Supplementary file 1 [file cancers-13-04910-s001.zip › cancers-1366162-tex.zip!/Definitions/logo-mdpi-eps-converted-to.pdf]

MDPI
